# DeLLITE Depression in late life: an intervention trial of exercise. Design and recruitment of a randomised controlled trial

**DOI:** 10.1186/1471-2318-8-12

**Published:** 2008-05-24

**Authors:** Ngaire Kerse, Karen Falloon, Simon A Moyes, Karen J Hayman, Tony Dowell, Gregory S Kolt, C Raina Elley, Simon Hatcher, Kathy Peri, Sally Keeling, Elizabeth Robinson, John Parsons, Janine Wiles, Bruce Arroll

**Affiliations:** 1 Department of General Practice and Primary Health Care, School of Population Health, Faculty of Medical and Health Sciences, University of Auckland, Auckland, New Zealand; 2 Department of General Practice, Wellington Clinical School, University of Otago, Wellington New Zealand; 3 School of Biomedical and Health Sciences, University of Western Sydney, Sydney, Australia; 4 Centre for Physical Activity and Nutrition Research, Auckland University of Technology, Auckland, New Zealand; 5 Department of Psychological Medicine, Faculty of Medical and Health Sciences, University of Auckland, Auckland, New Zealand; 6 School of Nursing. Faculty of Medical and Health Sciences, University of Auckland, Auckland, New Zealand; 7 Health Care of the Elderly, University of Otago, Christchurch, New Zealand; 8 Department of Epidemiology and Biostatistics. School of Population Health, Faculty of Medical and Health Sciences, University of Auckland, Auckland, New Zealand; 9 Department of Social and Community Health, School of Population Health, Faculty of Medical and Health Sciences, University of Auckland, Auckland, New Zealand

## Abstract

**Background:**

Physical activity shows potential in combating the poor outcomes associated with depression in older people. Meta-analyses show gaps in the research with poor trial design compromising certainty in conclusions and few programmes showing sustained effects.

**Methods/design:**

The Depression in Late Life: an Intervention Trial of Exercise (DeLLITE) is a 12 month randomised controlled trial of a physical activity intervention to increase functional status in people aged 75 years and older with depressive symptoms. The intervention involves an individualised activity programme based on goal setting and progression of difficulty of activities delivered by a trained nurse during 8 home visits over 6 months. The control group received time matched home visits to discuss social contacts and networks. Baseline, 6 and 12 months measures were assessed in face to face visits with the primary outcome being functional status (SPPB, NEADL). Secondary outcomes include depressive symptoms (Geriatric Depression Scale), quality of life (SF-36), physical activity (AHS Physical Activity Questionnaire) and falls (self report).

**Discussion:**

Due to report in 2008 the DeLLITE study has recruited 70% of those eligible and tests the efficacy of a home based, goal setting physical activity programme in improving function, mood and quality of life in older people with depressive symptomatology. If successful in improving function and mood this trial could prove for the first time that there are long term health benefit of physical activity, independent of social activity, in this high risk group who consume excess health related costs.

**Trial registration:**

Australian and New Zealand Clinical Trials Register ACTRN12605000475640

## Background

Depression is a common condition in older people, affecting between 17 and 30% of community dwellers over the age of 65 years [1]. Depression can be associated with significant disability, adverse health outcomes, poor quality of life and excess mortality [2] and thus is a significant concern for older people.

Treatment of depression in older people has limitations. Pharmaceutical interventions have potential side effects and potentiate drug interactions which can be particularly hazardous in older people. Psychological therapies [3] are limited by their availability in many settings, and for many therapies, the evidence base for their impact in elderly populations is lacking. Therefore, there is a need to develop and test new treatments and ways of enhancing existing treatments for depression in older people to prevent the morbidity and mortality afforded by this serious condition.

### Depression and physical activity

Recent randomised controlled trials and meta-analysis have demonstrated the antidepressant effect of physical activity and exercise in older adults with clinical depression [4-6]. Different forms of physical activity have been studied. While several trials have shown that progressive resistance training delivered over 10 weeks improves mood in the short term [7], other trials have tested the benefits of aerobic activity [4,5,8] and moderate activity such as walking [9].

The frequency of activity may be more important than the intensity of sessions and the longer the intervention is maintained the greater the benefit [10]. Both moderate and vigorous levels of activity show efficacy in reducing depressive symptoms [11] and progressive resistance training is equally as successful as aerobic activity [10].

Meta-analyses however have been critical of the quality of trials [12] with commonly identified difficulties being a lack of standard diagnostic process for identifying depression in participants, short follow up periods, control groups that do not control for the attention of the exercise facilitator and small effect sizes [11,12]. There are few programmes that suggest ease in widespread dissemination.

### Depression and functional status

The disability from depression has been shown to be greater than that from major chronic medical conditions [13-15]. The greater the depressive symptoms, the greater the decrease in level of function [16,17]. Functional limitation has been shown to predict a variety of adverse outcomes including depression, rest home admission and mortality [16,17] and higher rates of depression are associated with being home bound [18], socially isolated [19] and medically unfit [20].

Physical disability is also a risk factor for the development of depression [21] supporting the hypothesis that disability and depression are interrelated, creating a spiral of increasing psychological and physical decline [22]. Physical activity can alleviate the development of disability [23], however, sustainability is key and people with poor socialisation patterns and depression are difficult to engage in new activity programmes [24].

### Social Participation

Social participation and social support networks are paramount to long term positive outcomes and psychological wellbeing for older people [25,26]. A social context for physical activity and activity of a moderate rather than vigorous intensity promotes participation among older people [27]. Sedentary older people are more socially isolated than those participating in regular physical activity [28]. Continued support is important to interventions achieving long lasting change in exercise behaviour [29]. It is clear that social issues contribute to depression [30] and the importance of caregivers and other social supports in successful ageing for older people is well understood [31]. A social context to the physical activity intervention may be important to promote participation of older people. As depression is associated with poor participation in physical activity and health promotion programmes [32] increased effort in recruitment will be needed to engage older people with depression.

### Developing a programme

We adapted an existing exercise programme, the *Otago Exercise Programme (OEP)*, which has been shown to improve function and reduce the risk of falls among older adults [33]. The OEP is a set of individualised progressive lower leg strengthening and balance retraining exercises coupled with regular walking. We have enhanced the OEP regimen with two additional home visits and additional exercises focussed on upper limb strengthening. Two further adaptations were added to encourage participation and sustainability considering the target group (older people with depression) including goal setting and involvement of a social 'coach' or person to complete activities with the older person.

Increasing depressive symptoms lead to a decline in physical performance [16]. To improve function for people with depression is likely to enhance independence and improve quality of life for the participant. Whilst depression is a relevant outcome in itself, reducing the disability and dependency related to that diagnosis are key aims and outcome measures for this trial.

We sought to develop a sustainable programme designed to be delivered at home and test it in a rigorous trial addressing appropriate description of levels of depression at entry, using a control group receiving similar amounts of attention to control for the social impact of activity training and with long term follow up to establish potential long term effectiveness and sustainability of activity.

In this study we aimed to assess the impact in depressed older people of this home-based physical activity programme, incorporating goal setting, in improving functional status, depressive symptoms and quality of life. This paper describes the study protocol and recruitment for the Depression in Late Life: an Intervention Trial of Exercise (DeLLITE).

## Methods

### Design

A randomised controlled trial design was used to test the efficacy of a home based physical activity programme in improving health outcomes in depressed older people compared with a control group receiving a social intervention.

#### Participants

Community dwelling adults aged 75 years and older from Auckland, New Zealand, general practices were identified through practice-based computer systems. Exclusion criteria included: living in residential aged care, severe dementia, terminal illness, not able to communicate in English or unstable medical condition such that participation in a physical activity programme was unsafe (as judged by their general practitioner (GP)).

#### Recruitment (see Figure 1)

**Figure 1:**
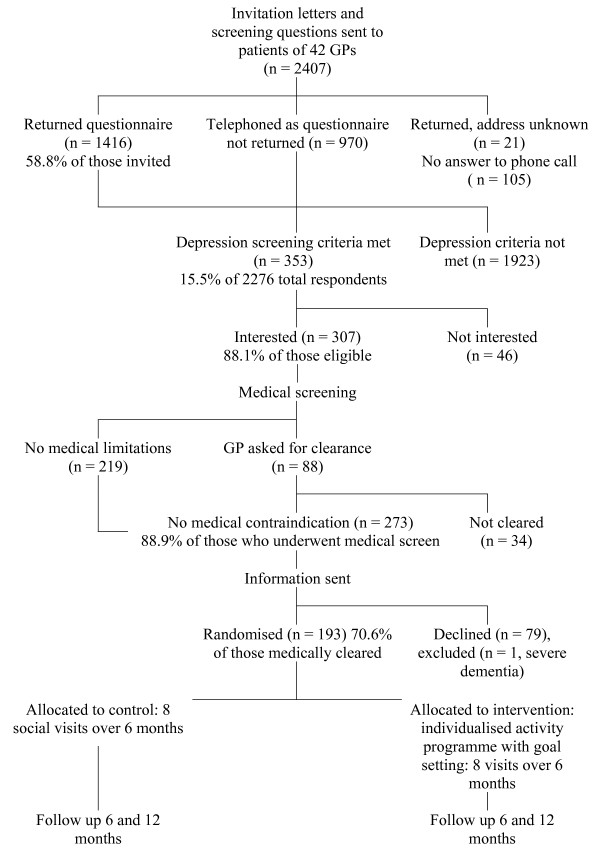
Recruitment Flowchart and Identification of Eligible Population.

A random sample of general practitioners from three large urban suburbs in the Auckland region were approached in random order with a faxed invitation. Interested doctors were visited by a research nurse, the study explained and a list of all patients 75 years and older generated. The GP and practice staff perused the list to exclude patients who were not community-dwelling, who had dementia or an unstable medical condition or who would not be able to participate in a physical activity programme. An invitation letter, signed by the GP was mailed to the patients with a brief depression screen comprising three questions [34]:

1) During the past month have you often been bothered by feeling down, depressed or hopeless?

2) During the past month have you often been bothered by having little interest or pleasure in doing things?

3) Answer if you responded 'yes' to 1. or 2: Is this something with which you would like help?

Scores of two yes's of a possible three were considered sufficient for inclusion. Completed questionnaires were returned in a reply paid envelope with an indication of whether the potential participant was interested in hearing more about the study. Those who did not send back the screening questionnaire were contacted by telephone by a researcher and the three question depression screen administered.

Those who were interested and who fulfilled criteria were phoned by a research nurse and asked the following medical questions:

1) Have you suffered from a heart attack in the past 6 months?

2) Have you suffered from a stroke in the past 6 months?

3) Do you suffer from:

a. Ongoing chest pains?

b. Dizziness?

c. Fainting attacks?

d. Shortness of breath that stops you from walking across the room?

If the potential participant 'failed' the medical screen, the researcher faxed the GP a request for medical clearance.

Recruitment processes were undertaken over 10 months from March 2006-January 2007 to allow the intervention to be delivered during all seasons as seasonal variation in mood status may impact results [35]. Written informed consent was obtained in person during a home visit.

#### Randomisation and Blinding

A researcher at a distant site allocated individuals to each group according to a computer-generated randomisation schedule. Couples were given the same allocation to avoid cross contamination. Assessors remained blind to allocation of randomisation at each measurement point.

#### Intervention Group

Intervention group participants were visited at home by a trained intervention nurse and instructed in an individualised home-based physical activity programme. The programme was based on the participant setting a functional goal (e.g. being able to prune the roses again or able to do a load of washing independently) and the nurse completing a functional assessment and designing an individualised programme based on the functioning needed for the goal [36]. The programme incorporated the activities of the Otago Exercise Programme[33] with regular walking three times per week, progressive resistance training of the upper and lower limbs using weights and balance retraining using progressive balance exercises. Adherence to activities was promoted by involvement of a companion, already known to the participant, so that walking was accompanied and exercises encouraged. The walking companion may have been a friend, relative, carer, or a community volunteer where this was available.

The intervention nurse made eight home visits, six in the first two months, a seventh at three months and a final visit at six months. To ensure adherence and progression of activity fridge calendars were used by the intervention group participants to record the number of exercise episodes and walking frequency. These were continued and either posted back or collected by the intervention nurse throughout the year of follow up to encourage and measure adherence.

#### Control Group

Control group participants received social visits from the social visitor with conversation based around current social activities and social networks. These visits were time-matched to the intervention group visits and eight visits were received in total over 6 months. Each visit took half to one hour. This was used to control for the potential social aspect of being visited by the intervention nurse. This amount of social contact has the potential to improve mood and we wished to establish the effect of activity independent of the social contact of the intervention nurse.

#### Outcome Measures *(See *Table 1)

**Table 1:** Outcome Measures

	**Outcome Measure**	**Description**	**Explanation**
**Primary**	Functional status – observed	Short Physical Performance Battery (SPPB) score [16, 17]	SPPB group and summary scores show a clear gradient. Lower scores correlate with higher mortality rates and nursing home admissions.
	Functional status- self report	Nottingham Extended Activities of Daily Living Scale (NEADL) score [37]	A well validated measure of self report functional status that is sensitive to change.

**Secondary**	Depressive symptoms	Geriatric Depression Scale (GDS-15) score	An extensively used and well validated scale [38]. It can be used both as a screening tool for depression and as a measure of change of symptoms [43, 44]. GDS-15 = 5 indicates significant depressive symptoms.
	Quality of life	SF-36 score [39]	This validated measure evaluates eight subscales of health-related quality of life.
	Physical Activity	Auckland Heart Study (AHS) Physical Activity Questionnaire score [40, 45].	Validated [45] self- report questionnaire.
	Falls and injuries	Self-reported falls and injuries in the last 12 months. Medical treatment for injuries from falls	This will help determine if falls are reduced or increased by the programme (ie: a potential harm).

The primary outcome measure is functional status measured using standardised tests. Both the Short Physical Performance Battery (SPPB) [16,17] and self-reported Nottingham Extended Activities of Daily Living (NEADL) [37] were used to measure functional status. Functional status measurements were administered by trained interviewers. Secondary outcome measures include depressive symptoms, the Geriatric Depression Scale (GDS-15) [38], health related quality of life, (SF-36) [39], physical activity (the Auckland Heart Study (AHS) Physical Activity Questionnaire) [40], and falls reported at follow up times. Baseline demographic information was collected by an interviewer-administered structured questionnaire. Medications were recorded by the research nurse on direct observation. Repeated blood pressures and physical performance tests were also conducted

All participants were visited in their own home and the outcome measures recorded at baseline, 6 (end of intervention) and 12 months. In addition to exercise calendars, a researcher carried out a follow up phone call at 12 months to retrospectively assess self-reported adherence.

#### Sample size

Based on means, standard deviations and change from previous trials, a sample of 70 per group would have at least 90% power at the 0.05 level of significance to detect a true difference in the change between the two groups of 2 points on the SPPB, 1 point on the Geriatric Depression Scale and 10 points in the Physical Component Score of the SF-36 [41]. To allow for an attrition rate of 25% over 12 months, 95 participants in each group were required.

### Analysis

Repeated measures analyses using generalised linear mixed models which take into account the covariance pattern of the data will be used to investigate changes over time in the major outcomes and whether these changes differ between the intervention and control groups. These models will be robust if data is missing at random [42]. Baseline measures and demographics of participants will be included as covariates in the models. For the final results, an intention to treat analysis will be undertaken. All analyses will be undertaken using SAS 9.1.3 (SAS Institute Cary NC, USA).

The Northern X Regional Ethics Committee approved the study.

## Results

### Recruitment

*(See *Figure 1) Forty-two of the 93 GPs in the 34 practices approached agreed to participate (45%). One hundred and ninety three participants were recruited from 15 urban general practices in the central Auckland region. This represents 60.9% (193/317) of those meeting inclusion criteria (eligible population). Of those who met depression criteria alone 35% did not agree to participation (125/353).

## Discussion

Screening for depression in a questionnaire was valid and feasible using the three question depression screen [34]. Almost complete ascertainment of eligibility was achieved in this population based sample. Those identified with depressive symptoms were open to participation with 70.6% of those cleared for participation willing to take part after having received the study information.

Results will be available in mid 2008. The study utilises a home-based setting for the delivery of an individualised physical activity programme. Should this activity programme increase functional status, improve mood and quality of life for depressed older people, their health services utilisation is likely to decrease, independence will be promoted and the burden on families and caregivers may be lighter. A reduction in disability related to functional limitations and an improvement in activity level will also benefit other areas of health including: cardiovascular health, glucose metabolism and falls. A prolonging of independence may reduce institutionalisation and the need for formal community support services. There is also a potential reduction in health spending as a result of a potential maintenance or increase in functional status and quality of life.

## Competing interests

The authors declare that they have no competing interests.

## Authors' contributions

NK conceived the study, contributed to design and methodology, supervised the overall study conduct, directed analyses and assisted in manuscript preparation. KF, ER, SAM assisted in study design and supervision, data management and analysis and preparation of the manuscript. KJH, CRE, GSK, KP, JP conceived and refined the activity intervention, contributed to overall design, and contributed to manuscript preparation. KJH managed the study processes. SK, JW, conceived the social intervention, contributed data collection and to study design and manuscript preparation, TD, SH, KP, BA, CRE contributed to outcomes selection, overall study processes and manuscript preparation. SH provided supervision to all study staff during the period of assessment and intervention delivery.

## Pre-publication history

The pre-publication history for this paper can be accessed here:



## References

[B1] Baldwin R, Chiu C, Katona C (2002). Guidelines on depression in older people – practicing the evidence.

[B2] Mor V, Murphy J, Masterson-Allen S, Willey C, Razmpour A, Jackson ME, Greer D, Katz S (1989). Risk of functional decline among well elders. J Clin Epidemiol.

[B3] Ciechanowski P, Wagner E, Schmaling K, Schwartz S, Williams B, Diehr P, Kulzer J, Gray S, Collier C, LoGerfo J (2004). Community-integrated home-based depression treatment in older adults: a randomized controlled trial. JAMA.

[B4] Blumenthal JA, Babyak MA, Moore KA, Craighead WE, Herman S, Khatri P, Waugh R, Napolitano MA, Forman LM, Appelbaum M (1999). Effects of exercise training on older patients with major depression. Arch Int Med.

[B5] Singh NA, Clements KM, Singh MA (2001). The efficacy of exercise as a long-term antidepressant in elderly subjects: a randomized, controlled trial. J Gerontol.

[B6] Taylor AH, Cable NT, Faulkner G, Hillsdon M, Narici M, Bij AK Van Der (2004). Physical activity and older adults: a review of health benefits and the effectiveness of interventions. J Sp Sci.

[B7] Singh N, Clements K, Fiatarone M (1997). A randomized controlled trial of progressive resistance training in depressed elders. J Gerontol A Biol Sci Med Sci.

[B8] Babyak M, Blumenthal JA, Herman S, Khatri P, Doraiswamy M, Moore K, Craighead WE, Baldewicz TT, Krishnan KR (2000). Exercise treatment for major depression: maintenance of therapeutic benefit at 10 months. Psychosom Med.

[B9] McNeil JK, LeBlanc EM, Joyner M (1991). The effect of exercise on depressive symptoms in the moderately depressed elderly. Psychol Aging.

[B10] Brosse AL, Sheets ES, Lett HS, Blumenthal JA (2002). Exercise and the treatment of clinical depression in adults: recent findings and future directions. Sp Med.

[B11] Dunn AL, Trivedi MH, O'Neal HA (2001). Physical activity dose-response effects on outcomes of depression and anxiety. Med Sci Sports Exerc.

[B12] Lawlor DA, Hopker SW (2001). The effectiveness of exercise as an intervention in the management of depression: systematic review and meta-regression analysis of randomised controlled trials. BMJ.

[B13] Wells KB, Burnam MA, Rogers W, Hays R, Camp P (1992). The course of depression in adult outpatients. Arch Gen Psychiatr.

[B14] Wells KB, Stewart A, Hays RD, Burnam MA, Rogers W, Daniels M, Berry S, Greenfield S, Ware J (1989). The functioning and well-being of depressed patients. Results from the Medical Outcomes Study. JAMA.

[B15] Moussavi S, Chatterji S, Verdes E, Tandon A, Patel V, Ustun B (2007). Depression, chronic diseases, and decrements in health: results from the World Health Surveys. Lancet.

[B16] Guralnik JM, Simonsick EM, Ferrucci L, Glynn RJ, Berkman LF, Blazer DG, Scherr PA, Wallace RB (1994). A short physical performance battery assessing lower extremity function: association with self-reported disability and prediction of mortality and nursing home admission. J Gerontol.

[B17] Guralnik JM, Ferrucci L, Simonsick EM, Salive ME, Wallace RB (1995). Lower-extremity function in persons over the age of 70 years as a predictor of subsequent disability. N Eng J Med.

[B18] Banerjee S (1993). Prevalence and recognition rates of psychiatric disorder in the elderly clients of a community care service. Int J Geriatr Psychiatry.

[B19] Prince MJ, Harwood RH, Thomas A, Mann AH (1998). A prospective population-based cohort study of the effects of disablement and social milieu on the onset and maintenance of late-life depression. Psycholog Med.

[B20] Geerlings SW, Beekman AT, Deeg DJ, Van Tilburg W (2000). Physical health and the onset and persistence of depression in older adults: an eight-wave prospective community-based study. Psycholog Med.

[B21] Bruce ML, Hoff RA (1994). Social and physical health risk factors for first-onset major depressive disorder in a community sample. Soc Psychiatry Psychiatr Epidemiol.

[B22] Bruce ML, Seeman TE, Merrill SS, Blazer DG (1994). The impact of depressive symptomatology on physical disability: MacArthur Studies of Successful Aging. Am J Pub Health.

[B23] Gill TM, Baker DI, Gottschalk M, Peduzzi PN, Allore H, Byers A (2002). A program to prevent functional decline in physically frail, elderly persons who live at home. N Eng J Med.

[B24] Watkins A, Kligman E (1993). Attendance patterns of older adults in a health promotion program. Public Health Reports.

[B25] Bowling A (1991). Social support and social networks: their relationship to the successful and unsuccessful survival of elderly people in the community. An analysis of concepts and a review of the evidence. Fam Pract.

[B26] Bowling A, Farquhar M (1991). Associations with social networks, social support, health status and psychiatric morbidity in three samples of elderly people. Soc Psychiatr Psychiatr Epidemiol.

[B27] King AC, Oman RF, Brassington GS, Bliwise DL, Haskell WL (1997). Moderate-intensity exercise and self-rated quality of sleep in older adults. A randomized controlled trial. JAMA.

[B28] Allison M, Keller C (1997). Physical activity in the elderly: benefits and intervention strategies. Nurse Pract.

[B29] Hillsdon M, Thorogood M, Anstiss T, Morris J (1995). Randomised controlled trials of physical activity promotion in free living populations: a review. J Epidemiol Community Health.

[B30] Mirza I, Jenkins R (2004). Risk factors, prevalence, and treatment of anxiety and depressive disorders in Pakistan: systematic review. BMJ.

[B31] Solomon DH, Wagner DR, Marenberg ME, Acampora D, Cooney LM, Inouye SK (1993). Predictors of formal home health care use in elderly patients after hospitalization. J Am Geriatr Soc.

[B32] McAuley E, Courneya KS, Rudolph DL, Lox CL (1994). Enhancing exercise adherence in middle-aged males and females. Prev Med.

[B33] Robertson M, Campbell A, Gardner M, al e (2002). Preventing injuries in elderly people by preventing falls: a meta-analysis of individual-level data. J Am Geriatr Soc.

[B34] Arroll B, Goodyear-Smith F, Kerse N, Fishman T, Gunn J (2005). Effect of the addition of a "help" question to two screening questions on specificity for diagnosis of depression in general practice: diagnostic validity study. BMJ.

[B35] Skegg K, Skegg DC, McDonald BW (1986). Is there seasonal variation in the prescribing of antidepressants in the community?. J Epidemiol Community Health.

[B36] Peri K, Kerse N, Robinson E, Parsons M, Parsons J, Latham N (2008). Does functionally-based activity make a difference to quality of life? A randomised controlled trial in residential care facilities. Age Ageing.

[B37] Essink-Bot ML, Krabbe PF, Bonsel GJ, Aaronson NK (1997). An empirical comparison of four generic health status measures. Medical Care.

[B38] Sheikh JJY (1986). Geriatric Depression Scale (GDS): Recent Evidence and Development of a Shorter Version. Clinical Gerontologist.

[B39] Brazier JE, Harper R, Jones NM, O'Cathain A, Thomas KJ, Usherwood T, Westlake L (1992). Validating the SF-36 health survey questionnaire: new outcome measure for primary care. BMJ.

[B40] Elley C, Kerse N, Swinburn B, Arroll B, Robinson E (2003). Measuring physical activity in primary health care research: Validity and reliability of two questionnaires. NZFP.

[B41] Ware J (1994). SF-36 Physical and Mental Health Summary Scales: a user's manual.

[B42] Brown H, Prescott R (1999). Applied Mixed Models in Medicine.

[B43] Gantner AB, Schubert DS, Wolf SR, Creps P (2003). Screening for depression in a geriatric rehabilitation sample. Int J Psychiatr Med.

[B44] Fabacher DA, Raccio-Robak N, McErlean MA, Milano PM, Verdile VP (2002). Validation of a brief screening tool to detect depression in elderly ED patients. Am J Emerg Med.

[B45] Jackson R (1989). The Auckland Heart Study: A case-control study of coronary heart disease [PhD].

